# Supplementary material for: Clout, activists and budget: The road to presidency
Source: PLoS One. 2018 Mar 1;13(3):e0193199. doi: 10.1371/journal.pone.0193199 (PMC5832241; doi:10.1371/journal.pone.0193199)
Supplement: S1 File — Additional information and mathematical proofs. (PDF) [file pone.0193199.s001.pdf]

Supporting Information: Clout, Activists and Budget: The Road to Presidency

L. Böttcher,[1,*] H. J. Herrmann,[1] and H. Gersbach[2]

[1]*ETH Zurich, Wolfgang-Pauli-Strasse 27, CH-8093 Zurich, Switzerland*
[2]*ETH Zurich, Zürichbergstrasse 18, CH-8092 Zurich, Switzerland*

(Dated: January 26, 2018)

* lucasb@ethz.ch

I. MODEL PROPERTIES

A. Recap of the model definition

Mean-field approximations have been proven useful to gain important insights about a given spreading dynamics [1, 2] and many real-world networks exhibit mean-field features [3]. Thus, we first shortly recapitulate the model definition to then discuss the properties of our mean-field approximation in the subsequent paragraphs. The model as defined in the main text describes the competition of two campaign groups A and B on a network with N nodes. Each campaign group consists of activists and persuadable individuals whose fractions are denoted by a^+, b^+ and a^0, b^0, respectively. A fraction o of nodes is empty and activists A (B) are able to occupy these empty places to convince neighboring persuadable ones with probability $\rho_A \in (0,1)$ ($\rho_B \in (0,1)$) if the utility condition $\rho_A \# B^0 - c_A > 0$ ($\rho_B \# A^0 - c_B > 0$) is satisfied and if enough budget $\mathcal{B}_A$ ($\mathcal{B}_B$) is available. The first condition corresponds to the situation where activists of campaign group A convince persuadable B^0 nodes if the product of convincing probability ρ_A and number of neighboring persuadable nodes $\#B^0$ is larger than the associated cost c_A. In the same manner, the second condition restricts the actions of activists of campaign group B. If an activist moves onto an empty node, the cost is deducted from the corresponding budget. We assume that the fractions of activists and empty nodes are constant whereas the fractions of persuadable nodes change over time. For analytical tractability, we also assume a *regular network with fixed degree* k, i.e. a fixed number of neighbors. A mean-field approach for general degree distributions f_k is presented in Refs. [4, 5]. Under the assumption of a perfectly mixed population of nodes in the thermodynamic limit, the dynamics is described by

$$\begin{aligned} \dot{a}^0(t) =& f_{B^0\to A^0}(t) - f_{A^0\to B^0}(t) \\ =& o\rho_A \Theta(\mathcal{B}_A) \sum_{j=\left\lceil \frac{c_A}{\rho_A} \right\rceil}^{k} \sum_{l,m.n,r \in S_{k-j}} j \binom{k}{j,l,m,n,r} {b^0}^j {a^+}^{l+1} {b^+}^m {a^0}^n o^r \\ & - o\rho_B \Theta(\mathcal{B}_B) \sum_{j=\left\lceil \frac{c_B}{\rho_B} \right\rceil}^{k} \sum_{l,m.n,r \in S_{k-j}} j \binom{k}{j,l,m,n,r} {a^0}^j {a^+}^{l} {b^+}^{m+1} {b^0}^n o^r, \end{aligned} \tag{S1}$$

where $\Theta(\cdot)$ denotes the Heaviside step function accounting for the fact that opinion changes only occur when enough budget is available and $\binom{k}{j,l,m,n,r}$ is the multinomial coefficient such that $j+l+m+n+r=k$ and $S_{k-j} = \{l,m,n,r \geq 0, l+m+n+r = k-j\}$. We introduced the transition functions $f_{B^0\to A^0}(t)$ and $f_{A^0\to B^0}(t)$ as a shorthand notation for the corresponding opinion changes. The dynamics of B^0 nodes is contained in Eq. (S1) since $b^0 = 1 - a^+ - b^+ - o - a^0$.

The cost deductions from the respective budgets are described by

$$\dot{\mathcal{B}}_A(t) = -\frac{c_A}{\rho_A} f_{B^0\to A^0}(t), \tag{S2}$$

$$\dot{\mathcal{B}}_B(t) = -\frac{c_B}{\rho_B} f_{A^0\to B^0}(t), \tag{S3}$$

with the initial conditions $\mathcal{B}_A(0) = \mathcal{B}_A^0$, $\mathcal{B}_B(0) = \mathcal{B}_B^0$. If not noted otherwise, we assume unlimited resources ($\Theta(\mathcal{B}_A) = 1$ and $\Theta(\mathcal{B}_B) = 1$ for all times t) in the subsequent sections to characterize the stationary states of Eq. (S1). In the case of limited resources, the activist dynamics would just stop in one or the other campaign group.

B. Uniqueness of the stationary solution

Is there always a unique stationary solution of Eq. (S1), i.e. a unique stationary $a^0_{\rm st}$ and $b^0_{\rm st}$ in the interval $\left(0, a^0(0) + b^0(0)\right]$? We remember that the fractions of activists a^+, b^+ and of empty nodes o are constant over time. Thus, the dynamics only applies to persuadable nodes leading to trajectories where $a^0(t),\ b^0(t) \in \left(0, a^0(0) + b^0(0)\right]$. In Fig. 1 (left) we show a typical time evolution of our dynamics. The corresponding phase portrait is illustrated in Fig. 1 (right). We clearly see that there is only one stable fixed point for a^0 and b^0.

To obtain some insights into the general behavior of the stationary solutions, we simplify Eq. (S1) by solely focusing on the dynamical part of the network. More specifically, we introduce an effective degree $k_{\rm eff} = \lceil k(1 - a^+ - b^+ - o) \rceil$ and an effective threshold $m_{\rm eff} = \left\lceil \frac{c}{\rho}(1 - a^+ - b^+ - o) \right\rceil$, assuming $\rho_A = \rho_B = \rho$ and $c_A = c_B = c$. Approximating

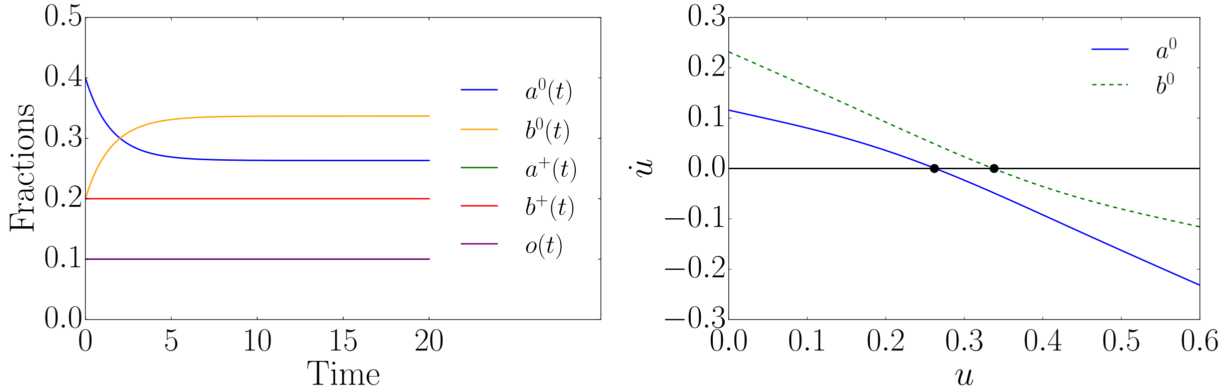

Figure S1. **Time evolution and phase portrait of the campaign model.** The shown curves are a numerical solution of Eq. (S1) for $k = 10$ with initial conditions $a^0(0) = 0.4$, $b^0(0) = 0.2$, $a^+(0) = 0.1$, $b^+(0) = 0.2$ and $o(0) = 0.1$. The convincing probability is set to $\rho_A = \rho_B = 0.5$ and the costs are $c_A = c_B = 2$. The left panel displays the time evolution of the fractions of persuadable nodes $a^0(t)$, $b^0(t)$ and activists $a^+(t)$, $b^+(t)$ as well as empty nodes $o(t)$. In the right panel, the corresponding phase portrait is shown. Black dots represent stable fixed points.

Eq. (S1) by introducing an effective degree k_{eff} means to distribute the fractions a^0 and b^0 of nodes in a network with degree k_{eff}. An effective threshold m_{eff} can be seen as the consequence of a reduced cost due to the reduced number of neighbors. We discuss the general case of two thresholds m_{eff}^A and m_{eff}^B subsequently. In the following equation we interpret x as the fraction a^0 and $1-x$ as the fraction b^0.

$$\dot{x} = \sum_{j=m_{\text{eff}}}^{k_{\text{eff}}} j \binom{k_{\text{eff}}}{j} \left[\lambda \left(1-x\right)^j x^{k_{\text{eff}}-j} - x^j \left(1-x\right)^{k_{\text{eff}}-j} \right], \tag{S4}$$

where $\lambda = a^+/b^+$. All other constant values have been neglected to increase readability and to ensure analytical tractability. Equation (S4) is an approximation of the time evolution of a^0 and b^0 as described by Eq. (S1). It is taking into account the model's important features and thus providing a framework to better understand the properties of the stationary solution.

We want to find out if the function has only one fixed point. Our first observation is that the right-hand side of Eq. (S4) defined as $f(x; m_{\text{eff}}, k_{\text{eff}}, \lambda)$ approaches $f(x; m_{\text{eff}}, k_{\text{eff}}, \lambda) = k_{\text{eff}}\lambda$ for $x \to 0$ and $f(x; m_{\text{eff}}, k_{\text{eff}}, \lambda) = -k_{\text{eff}}$ for $x \to 1$. If the function $f(x; m_{\text{eff}}, k_{\text{eff}}, \lambda)$ monotonically decreased in x in the interval $(0,1)$, there would be only a unique fixed point. For $m_{\text{eff}} = 0, 1$, the derivative with respect to x is given by $f'(x; m_{\text{eff}}, k_{\text{eff}}, \lambda) = -k_{\text{eff}}(1+\lambda)$ and for $m_{\text{eff}} = k_{\text{eff}}$ the derivative is $f'(x; m_{\text{eff}}, k_{\text{eff}}, \lambda) = -k_{\text{eff}}^2 \left[x^{k_{\text{eff}}-1} + \lambda \left(1-x\right)^{k_{\text{eff}}-1} \right]$. In both cases, the derivative indicates a monotonically decreasing function and thus the existence of a unique fixed point.

For a general threshold m_{eff} the derivative can be obtained using *Mathematica* [6],

$$\begin{aligned} f'(x; m_{\text{eff}}, k_{\text{eff}}, \lambda) = &- \left(\Gamma(k_{\text{eff}} + 1 - m_{\text{eff}})\Gamma(m_{\text{eff}}+1)\right)^{-1} k_{\text{eff}}((1-x)x)^{-m_{\text{eff}}-1}\Gamma(k_{\text{eff}}) \\ &\left[(1-x)^{2m_{\text{eff}}} x^{k_{\text{eff}}+1} \lambda \left(m_{\text{eff}}(m_{\text{eff}} - 1 + x) + k_{\text{eff}}(1-x)\, {}_2F_1\left(1, m - k_{\text{eff}};\, m_{\text{eff}}+1;\, \frac{x-1}{x}\right)\right) + \right. \\ &\left. (1-x)^{k_{\text{eff}}+1} x^{2m_{\text{eff}}} \left(m_{\text{eff}}(m_{\text{eff}} - x) + k_{\text{eff}} x\, {}_2F_1\left(1, m_{\text{eff}} - k_{\text{eff}};\, m_{\text{eff}}+1;\, \frac{x}{x-1}\right)\right)\right]. \end{aligned} \tag{S5}$$

Since x takes values in the interval $(0,1)$ and $m_{\text{eff}} \in \{2, \ldots, k_{\text{eff}}-1\}$, we only have to verify if the hypergeometric functions ${}_2F_1$ are positive. Then $f'(x; m_{\text{eff}}, k_{\text{eff}}, \lambda)$ would be negative. Due to the fact that $m_{\text{eff}} - k_{\text{eff}} < 0$, the hypergeometric function takes the form [6],

$${}_2F_1\left(1, m_{\text{eff}} - k_{\text{eff}};\, m_{\text{eff}}+1;\, \frac{x}{x-1}\right) = \sum_{n=0}^{k_{\text{eff}}-m_{\text{eff}}} (-1)^n \binom{k_{\text{eff}} - m_{\text{eff}}}{n} \frac{(1)_n}{(m_{\text{eff}}+1)_n} \left(\frac{x}{x-1}\right)^n, \tag{S6}$$

where $(\cdot)_n$ denotes the Pochhammer symbol. The last term $\frac{x}{x-1}$ is smaller than 0 and thus the terms of the sum are

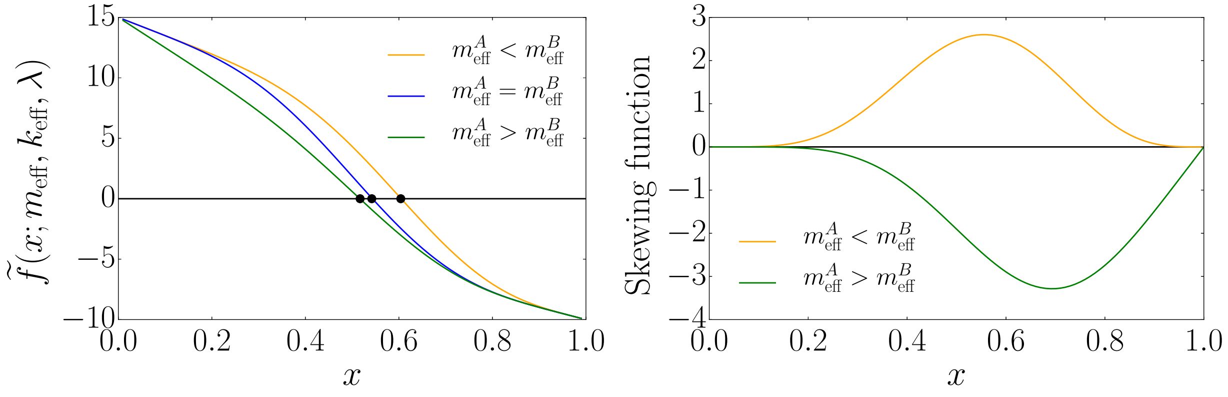

Figure S2. **General phase portrait and skewing functions.** The left panel shows the three possible cases of the campaign model's phase portrait where $\widetilde{f}\left(x; m^A_{\text{eff}}, m^B_{\text{eff}}, k_{\text{eff}}, \lambda\right)$ defines the right-hand side of Eq. (S8). We set $k_{\text{eff}} = 10$, $\lambda = 1.5$ and $m^A_{\text{eff}} = m^B_{\text{eff}} = 5$ (blue solid line) whereas $m^A_{\text{eff}} = 5 < m^B_{\text{eff}} = 7$ (orange solid line) and $m^A_{\text{eff}} = 5 > m^B_{\text{eff}} = 0$ (green solid line). In the right panel, we define the second sums of Eqs. (S9) and (S10) as the skewing functions that skew the monotonically decreasing first sums in positive ($m^A_{\text{eff}} < m^B_{\text{eff}}$) and negative ($m^A_{\text{eff}} > m^B_{\text{eff}}$) y-direction, respectively.

positive, since

$$ {}_2F_1\left(1, m_{\text{eff}} - k_{\text{eff}};\, m_{\text{eff}} + 1;\, \frac{x}{x-1}\right) = \sum_{n=0}^{k_{\text{eff}} - m_{\text{eff}}} \binom{k_{\text{eff}} - m_{\text{eff}}}{n} \frac{(1)_n}{(m_{\text{eff}} + 1)_n} \left(\frac{x}{1-x}\right)^n . \tag{S7} $$

The same argument applies to ${}_2F_1\left(1, m_{\text{eff}} - k_{\text{eff}};\, m_{\text{eff}} + 1;\, \frac{x-1}{x}\right)$. Therefore, $f'(x; m_{\text{eff}}, k_{\text{eff}}, \lambda) < 0$ and the basic model as defined by Eq. (S4) has one unique stationary state. We now focus on the more general case with two thresholds, i.e. $m^A_{\text{eff}} = \left\lceil \frac{c_A}{\rho_A} \right\rceil (1 - a^+ - b^+ - o)$, $m^B_{\text{eff}} = \left\lceil \frac{c_B}{\rho_B} \right\rceil (1 - a^+ - b^+ - o)$ and $\lambda = (\rho_A a^+) / (\rho_B b^+)$. Thus, the resulting dynamics is described by

$$ \dot{x} = \sum_{j=m^A_{\text{eff}}}^{k_{\text{eff}}} j \binom{k_{\text{eff}}}{j} \lambda \, (1-x)^j \, x^{k_{\text{eff}} - j} - \sum_{j=m^B_{\text{eff}}}^{k_{\text{eff}}} j \binom{k_{\text{eff}}}{j} x^j \, (1-x)^{k_{\text{eff}} - j} . \tag{S8} $$

Again, the right-hand side of Eq. (S8) approaches $\widetilde{f}\left(x; m^A_{\text{eff}}, m^B_{\text{eff}}, k_{\text{eff}}, \lambda\right) = k_{\text{eff}} \lambda$ as $x \to 0$ and $\widetilde{f}\left(x; m^A_{\text{eff}}, m^B_{\text{eff}}, k_{\text{eff}}, \lambda\right) = -k_{\text{eff}}$ as $x \to 1$. In order to show that $\widetilde{f}\left(x; m^A_{\text{eff}}, m^B_{\text{eff}}, k_{\text{eff}}, \lambda\right)$ decreases monotonically in x in the interval $(0, 1)$, we apply the previous proof by discussing the two cases: (i) $m^A_{\text{eff}} < m^B_{\text{eff}}$ and (ii) $m^A_{\text{eff}} > m^B_{\text{eff}}$. We find

$$ \text{(i)}\ \dot{x} = \sum_{j=m^A_{\text{eff}}}^{k_{\text{eff}}} j \binom{k_{\text{eff}}}{j} \left[\lambda \, (1-x)^j \, x^{k_{\text{eff}} - j} - x^j \, (1-x)^{k_{\text{eff}} - j}\right] + \sum_{j=m^A_{\text{eff}}}^{m^B_{\text{eff}} - 1} j \binom{k_{\text{eff}}}{j} x^j \, (1-x)^{k_{\text{eff}} - j} , \tag{S9} $$

$$ \text{(ii)}\ \dot{x} = \sum_{j=m^B_{\text{eff}}}^{k_{\text{eff}}} j \binom{k_{\text{eff}}}{j} \left[\lambda \, (1-x)^j \, x^{k_{\text{eff}} - j} - x^j \, (1-x)^{k_{\text{eff}} - j}\right] - \sum_{j=m^B_{\text{eff}}}^{m^A_{\text{eff}} - 1} j \binom{k_{\text{eff}}}{j} \lambda \, (1-x)^j \, x^{k_{\text{eff}} - j} . \tag{S10} $$

The first sums in Eqs. (S9) and (S10) decrease monotonically in x in the interval $(0, 1)$ as shown above for Eq. (S4). The second sums in Eqs. (S9) and (S10) approach 0 for $x = 0$ and $x = 1$ and otherwise only have positive values in the interval $(0, 1)$. We show two examples of these skewing functions in Fig. S2 (right). Consequently, as shown in Fig. S2 (left), the function defined by the first sum of Eq. (S9) gets skewed in positive y-direction without changing the fact that it decreases monotonically. In the case of Eq. (S10), the skewing occurs in negative y-direction, cf. Fig. S2 (left). Thus, there is still a unique stationary fixed point even for general thresholds that occur due to different ρ_A, ρ_B and c_A, c_B.

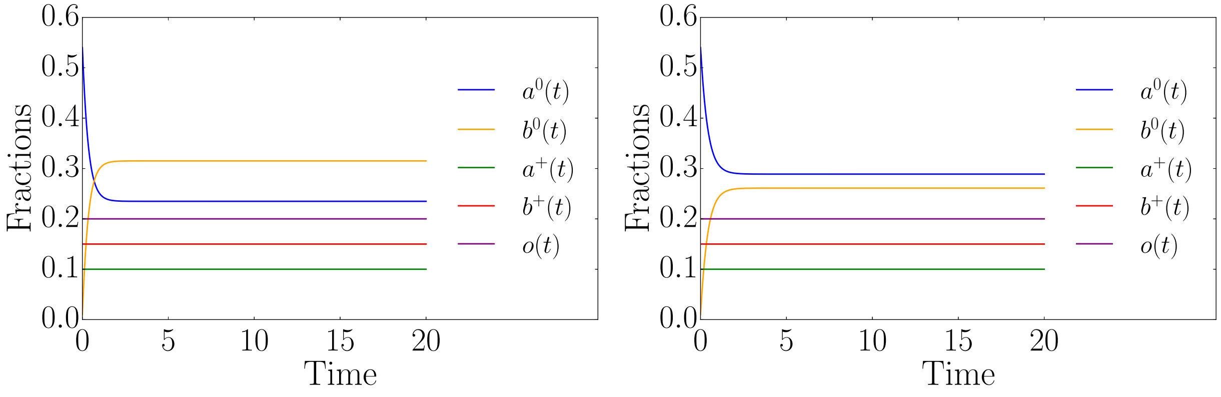

Figure S3. **Influence of activists and thresholds on the stationary states.** The time evolution of the fractions of persuadable nodes $a^0(t)$, $b^0(t)$ and activists $a^+(t)$, $b^+(t)$ as well as empty nodes $o(t)$ for two different parameter constellations are displayed. The shown curves are a numerical solution of Eq. (S1) for $k = 10$ with initial conditions $a^0(0) = 0.54$, $b^0(0) = 0.01$, $a^+(0) = 0.1$, $b^+(0) = 0.15$ and $o(0) = 0.2$. In the left panel, the convincing probabilities are $\rho = \rho_A = \rho_B = 1.0$ and the costs are set to $c = c_A = c_B = 2$. Group B, with the larger fraction of activists, dominates the dynamics. In the right panel, the convincing probabilities are $\rho_A = 1.0$ and $\rho_B = 0.8$, whereas the costs are still $c = c_A = c_B = 2$. Group A^0 dominates due to the larger convincing probability.

C. Interplay between activists, convincing probabilities and cost

Intuitively, the campaign with the larger fraction of activists and the larger convincing probability should be dominant in the stationary state. As in Sec. I B, we begin the discussion by setting $\rho = \rho_A = \rho_B$, $c = c_A = c_B$. Some arguments of the model's behavior for general ρ_A, ρ_B, c_A, c_B are given subsequently. In Fig. S1 we see that the stationary state of campaign group B is larger than the one of group A, since $b^+ > a^+$. Can this behavior be observed independently of the initial conditions? This would imply that only activists matter. We illustrate a solution of Eq. (S1) with $a^+(0) = 0.1$, $b^+(0) = 0.15$, $a^0(0) = 0.54$, $b^0(0) = 0.01$, $o(0) = 0.2$ in Fig. S3. As in Fig. S1, we again find that the group with more activists dominates the dynamics. We can also give some analytical insight into this behavior. For the moment, we assume that $\left\lceil \frac{c}{\rho} \right\rceil = k$ in Eq. (S1). Then $\dot{a} = o\rho k \left(a^+ {b^0}^k - b^+ {a^0}^k \right)$ and the corresponding stationary state reveals that $\left(\frac{a^0}{b^0}\right)^k = \left(\frac{a^+}{b^+}\right)$ in agreement with the expected influence of the activists as discussed above.

For general $\left\lceil \frac{c}{\rho} \right\rceil$, we find that the stationary states are equal if $a^+ = b^+$ due to the symmetric form of Eq. (S1). We now assume that $a^+ > b^+$ and focus on Eq. (S4) as before. The equation describing the stationary state x_{st} is given by

$$0 = \sum_{j=m_{\text{eff}}}^{k_{\text{eff}}} j \binom{k_{\text{eff}}}{j} \left[\lambda \left(\frac{1 - x_{\text{st}}}{x_{\text{st}}} \right)^j - \left(\frac{1 - x_{\text{st}}}{x_{\text{st}}} \right)^{k_{\text{eff}} - j} \right]. \tag{S11}$$

We know that there exists a unique solution $x_{\text{st}} \in (0, 1)$ as analytically demonstrated in Sec. I B. The solution $x_{\text{st}} = 0.5$ corresponds to the case where $a^+ = b^+$. We now prove by contradiction that $x_{\text{st}} > 0.5$ if $a^+ > b^+$, i.e. $\lambda > 1$. Hence, we assume that $x_{\text{st}} < 0.5$ if $\lambda > 1$. The term $\frac{1-x_{\text{st}}}{x_{\text{st}}} > 1$ and also $\sum_{j=m_{\text{eff}}}^{k_{\text{eff}}} j \binom{k_{\text{eff}}}{j} \left[\lambda \left(\frac{1-x_{\text{st}}}{x_{\text{st}}} \right)^j - \left(\frac{1-x_{\text{st}}}{x_{\text{st}}} \right)^{k_{\text{eff}}-j} \right] > 0$, since $\lambda > 1$. The negative terms appearing for $j < k_{\text{eff}} - j$ are always compensated by positive ones due to the summation over $j \in \{m_{\text{eff}}, \ldots, k_{\text{eff}}\}$, and the sum would not add up to 0. Thus, $x_{\text{st}} < 0.5$ cannot be a stationary state corresponding to $\lambda > 1$. Consequently, $x > 0.5$ if $a^+ > b^+$. We conclude that the campaign with the larger fraction of activists exhibits a dominating stationary state if convincing probability and costs are the same.

In the case of different convincing probabilities and costs, we end up with two different effective thresholds m_{eff}^A, m_{eff}^B and $\lambda = (\rho_A a^+) / (\rho_B b^+)$ as visible in Eqs. (S9) and (S10). The discussion of the latter two equations in Sec. I B revealed that additional sums just skew the monotonically decreasing right-hand sides in positive ($m_{\text{eff}}^A < m_{\text{eff}}^B$) and negative ($m_{\text{eff}}^A > m_{\text{eff}}^B$) y-direction respectively. This explains the influence of convincing probabilities and costs on the stationary states. A skewing in positive y-direction in the case of $m_{\text{eff}}^A < m_{\text{eff}}^B$ means that the threshold $m_{\text{eff}}^A = \left\lceil \frac{c_A}{\rho_A} \right\rceil (1 - a^+ - b^+ - o)$ is smaller than m_{eff}^B due to smaller cost and a larger convincing probability. Then

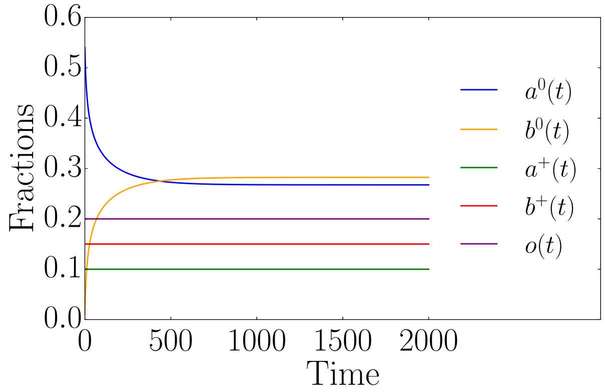

Figure S4. **Influence of costs and convincing probability.** The time-evolution of the fractions of persuadable nodes $a^0(t)$, $b^0(t)$ and activists $a^+(t)$, $b^+(t)$ as well as empty nodes $o(t)$ is displayed. The shown curves are a numerical solution of Eq. (S1) for $k = 10$ with initial conditions $a^0(0) = 0.54$, $b^0(0) = 0.01$, $a^+(0) = 0.1$, $b^+(0) = 0.15$ and $o(0) = 0.2$. The utility is fixed to $\rho = \rho_A = \rho_B = 0.5$ and the costs are $c = c_A = c_B = 4$.

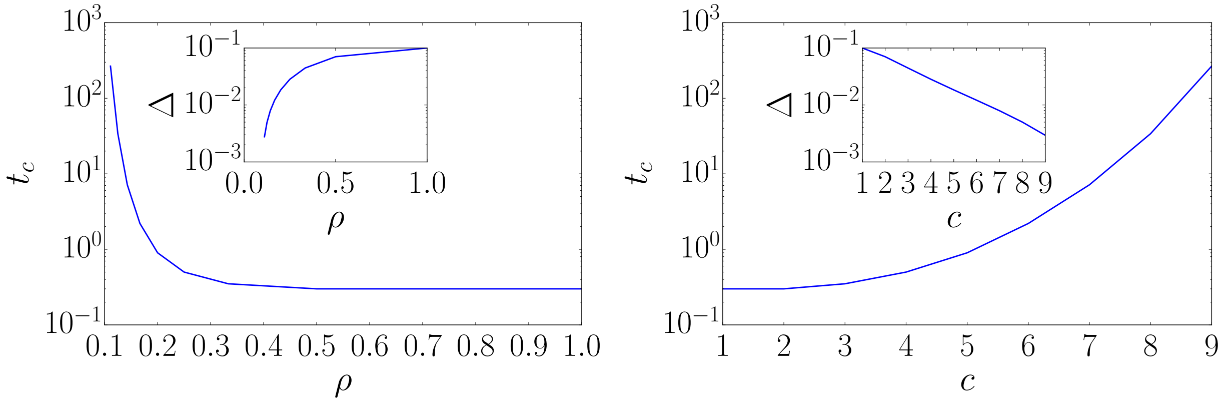

Figure S5. **Characteristic timescales and opinion gap.** The left panel show that a characteristic transient time-scale t_c decreases as $\rho = \rho_A = \rho_B$ increases The gap $\Delta = |b^0_{\text{st}} - a^0_{\text{st}}|$ between both stationary states, however, increases with ρ. The cost is set to $c = c_A = c_B = 1$. From the right panel, we find that the characteristic time scale t_c increases with the cost c, whereas the gap Δ decreases with c, as illustrated in the inset. The convincing probability has been fixed to $\rho = 1$. We numerically solved Eq. (S1) for $k = 10$, with $a^0(0) = 0.54$, $b^0(0) = 0.01$, $a^+(0) = 0.1$, $b^+(0) = 0.15$ and $o(0) = 0.2$.

the skewing in positive y-direction means that the final stationary state of campaign group A is getting larger, i.e. more individuals follow opinion A. In the same manner, the case where $m^A_{\text{eff}} > m^B_{\text{eff}}$ corresponds to a growing influence of campaign group B. In summary, there is a complex interplay between the fractions of activists, convincing probabilities and costs.

D. Further effects resulting from the influence of convincing probability and cost

In the previous Sec. I C, we have seen that not only the fractions of activists matter but that a change in convincing probability and cost also shifts the stationary states to the advantage or disadvantage of one campaign group. There are, however, other effects originating from the influence of convincing probability and costs that shall be discussed here. To properly analyze this influence and not to deal with too many parameters, we again set $\rho = \rho_A = \rho_B$ and $c = c_A = c_B$.

We first want to discuss the influence of the parameter ρ on the saturation time. The parameter ρ corresponds to a time-rescaling, since the increment of Eq. (S1), similar to $\dot{x} = \rho f(x)$, comes with a prefactor ρ. Smaller values of ρ would intuitively lead to longer transient times. Furthermore, ρ also enters the threshold condition of Eq. (S1) whose summation starts at $\left\lceil \frac{c}{\rho} \right\rceil$. We remember that no dynamics occurs if $\left\lceil \frac{c}{\rho} \right\rceil > k$ since this would imply that the number of persuadable nodes has to be greater than the actual degree of the network. Also in terms of the threshold

condition, we would expect longer transient times for smaller values of ρ. Larger threshold values correspond to the situation where activists require a greater number of persuadable nodes in order to perform an action, cf. Fig. S4. We illustrate the dependence of a characteristic transient time-scale t_c on ρ in Fig. S5 (left). In agreement with the qualitative arguments given above, we find that t_c decreases as ρ increases. Moreover, the inset in Fig. S5 (left) shows that the gap $\Delta = |b^0_{\mathrm{st}} - a^0_{\mathrm{st}}|$ between the stationary states increases with ρ.

Similar to the effect of a small convincing probability ρ, a large cost parameter c would intuitively lead to longer transients as shown in Fig. S4. Not all neighborhoods fulfill the utility condition in the case of a large cost and consequently it takes more time to reach the equilibrium. We also illustrate this behavior in Fig. S5 (right). As the cost parameter c increases, the characteristic time-scale t_c is getting larger. Moreover, we see in the inset of Fig. S5 (right) that the gap Δ between the two stationary opinions shrinks as the cost increases.

The following example illustrates an interesting effect occurring as a consequence of the shrinking gap for small values of ρ or large values of c. First, we compare Figs. S3 (left) and S4. We find that $a^0_{\mathrm{st}} \approx 0.235$ and $b^0_{\mathrm{st}} \approx 0.315$ in Fig. S3 (left), where $c = 2$ and $\rho = 1$. These values correspond to a gap of $\Delta = 0.08$. In Fig. S4 with $c = 4$ and $\rho = 0.5$ we, however, find a smaller gap of $\Delta = 0.014$, since $a^0_{\mathrm{st}} \approx 0.268$ and $b^0_{\mathrm{st}} \approx 0.282$. Taking into account the fractions of activists in both cases, i.e. $a^+ = 0.1$ and $b^+ = 0.15$, we conclude that 58 % are in favor of opinion B in the first case whereas this value is reduced to 54 % in the second case. In addition, we could think about a change of the majority structure as a consequence of the influence of ρ and c. We assume another compartment of inactive individuals with a fixed opinion a^- and b^-. They are not relevant for the dynamics at all but influence the majority structure. We now set $a^- = 0.1$ and $b^- = 0.02$, thereby implicitly reducing the value of o. As a consequence, 53 % of the individuals are in favor of opinion B in the first case but in the second case, this campaign group loses its majority with 49 %.

[1] M. J. Keeling and P. Rohani, *Modeling Infectious Diseases in Humans and Animals* (Princeton University Press, 2008).
[2] J. Marro and R. Dickman, *Nonequilibrium Phase Transitions in Lattice Models* (Cambridge University Press, 2005).
[3] J. P. Gleeson, S. Melnik, J. A. Ward, M. A. Porter, and P. J. Mucha, Phys. Rev. E **85**, 026106 (2012).
[4] L. Böttcher, M. Luković, J. Nagler, S. Havlin, and H. J. Herrmann, Sci. Rep. **7**, 41729 (2017).
[5] L. Böttcher, J. Nagler, and H. J. Herrmann, Phys. Rev. Lett. **118**, 088301 (2017).
[6] Wolfram Research, Inc., "Mathematica, Version 11.1," Champaign, IL, 2017.
